# We Really Need Clear Guidelines and Recommendations for Safer and Proper Use of Aripiprazole and Risperidone in a Pediatric Population: Real-World Analysis of EudraVigilance Database

**DOI:** 10.3389/fpsyt.2020.550201

**Published:** 2020-12-02

**Authors:** Concetta Rafaniello, Maria Giuseppa Sullo, Carla Carnovale, Marco Pozzi, Barbara Stelitano, Sonia Radice, Renato Bernardini, Francesco Rossi, Emilio Clementi, Annalisa Capuano

**Affiliations:** ^1^Section of Pharmacology “L. Donatelli”, Department of Experimental Medicine, Campania Regional Centre for Pharmacovigilance and Pharmacoepidemiology, University of Campania “Luigi Vanvitelli”, Naples, Italy; ^2^Unit of Clinical Pharmacology, Department of Biomedical and Clinical Sciences L. Sacco, “Luigi Sacco” University Hospital, Università di Milano, Milan, Italy; ^3^Scientific Institute Istituto di Ricovero e Cura a Carattere Scientifico-IRCCS E. Medea, Bosisio Parini, Italy; ^4^Unit of Clinical Toxicology, Department of Biomedical and Biotechnological Sciences, University Hospital, Catania, Italy

**Keywords:** Aripiprazole, risperidone, safety, pharmacovigilance, spontaneous reporting system, adverse drug reaction

## Abstract

**Background:** Although aripiprazole and risperidone are used widespread in pediatrics, there are still limited pieces of evidence on their actual safety profile. By using the EudraVigilance database, we carried out an analysis to perform a comprehensive overview of reported adverse events among children and adolescents treated with aripiprazole and risperidone.

**Methods:** Descriptive analysis was performed of all individual case safety reports (ISCRs) submitted to EudraVigilance associated with aripiprazole and risperidone and related to the pediatric population from 2016 to 2018.

**Results:** A total of 855 and 2,242 ISCRs for aripiprazole and risperidone, respectively, were recorded for a total of 11,042 suspected adverse drug reactions (2,993 for aripiprazole and 8,049 for risperidone). Most ISCRs were related to male patients (65.0 and 86.3% for aripiprazole and risperidone, respectively) and were serious (81.0 and 94.1% for aripiprazole and risperidone, respectively). Schizophrenia spectrum and other psychotic disorders, such as disruptive, impulse-control, and conduct disorders, and autism spectrum disorder were the top three clinical indications for aripiprazole (19.0, 16.1, and 11.6%, respectively). For risperidone, attention-deficit/hyperactivity disorder (25.4%), disruptive, impulse-control, and conduct disorders (17.1%), and bipolar and related disorders (14.2%) were more commonly reported as clinical indications. Data also showed a high proportion of use for clinical conditions not authorized in children. Psychiatric disorders were the main related adverse events for aripiprazole (20.2%), and among these, suicidal behavior was one of the most reported (14.9%). Reproductive system and breast disorders were the main related adverse events for risperidone (19.8%), and gynecomastia was the most reported event; metabolism and nutrition disorders, mainly reported as weight gain disorders, were more reported in children (3–11 years) than in adolescents (12–17 years).

**Conclusions:** Our results demonstrate that spontaneously reported adverse events associated with aripiprazole and risperidone reflect what is already known in terms of safety profile, although with about 90% of them being serious. This analysis stresses the need for further studies and effective training and information activities to better define the actual benefit/risk ratio of these drugs in pediatric patients.

## Introduction

Second-generation antipsychotics (SGAs) are becoming an increasingly integral part of comprehensive treatment programs of psychiatric disorders of childhood and adolescence. Over the last decade, several studies have indicated a substantial growth in the use of SGAs among children and adolescents, also reflecting their increasing off-label use (1).

Within the class of SGAs, risperidone, and aripiprazole are the most frequently prescribed in children and adolescents across most countries (2). They are approved medications for bipolar mania in children and adolescents, for adolescent schizophrenia, and for behavioral disturbances (irritability and aggression) associated with autism and/or intellectual disabilities in children and adolescents (3). Risperidone and aripiprazole are frequently prescribed in these populations also as treatment of attention-deficit/hyperactivity disorder (ADHD), eating disorders, anxiety, and sleep disorders (4, 5). Despite such a significant increase in SGA use in children and adolescents, reliable and comprehensive efficacy and safety information from specific clinical studies in pediatric patients are still relatively scant (4–6). The lack of information on efficacy and safety of these medications in pediatric settings indeed increases the exposure of this young population to unwarranted risks (7–10).

The adverse effects of SGAs range from relatively mild tolerability issues (e.g., mild sedation or dry mouth) to very unpleasant (e.g., constipation, akathisia, sexual dysfunction, acute dystonias, weight gain, or tardive dyskinesia) even up to life-threatening (e.g., myocarditis or agranulocytosis) (11). Moreover, because patients affected by specific psychiatric conditions may be more at risk of adverse effects, and each antipsychotic medication has a unique adverse effect profile (which affects individuals differently), the specific drug safety profile should be taken into account in treatment decision-making (3, 12). For example, an adolescent with autism and/or intellectual disabilities may be more at risk of adverse reactions due to neuro-developmental vulnerabilities, which in turn would further impair the child's developmental, social functioning, and challenging behavior leading to longer treatment duration (13); somnolence and sedation effects were frequently reported with the use of risperidone and aripiprazole in these patients, with a consequent impact on learning (3). Moreover, weight gain related to SGAs use in the pediatric population seems to be more frequently observed with risperidone than aripiprazole, although recent studies have demonstrated that both drugs, especially when used long-term at least for 12 months of therapy, lead to similar and considerable, lead to a significant weight gain (14). Why safety in the use of SGAs in pediatric settings remains still insufficiently addressed is likely due to a wide spectrum of complex aspects, e.g., the in/off label use of SGAs and the role of genetic factors together with biological and psychological differences among the pediatric age groups (15). Although studies have investigated about specific adverse drug reactions (ADRs) in pediatric settings, these were limited in their scope and rather focused on particular substances, specific countries, and/or selected subpopulations. No detailed pharmacovigilance studies based on large reporting system databases have been carried out, although this type of studies represents a valuable source to obtain a comprehensive overview and to gain knowledge on the safety of SGA drug use in a specific pediatric age group.

By using one of the largest and most comprehensive pharmacovigilance databases, i.e., EudraVigilance (EV), we carried out an analysis during the period 2016–2018 to perform a comprehensive overview of reported ADRs among a wide cohort of adolescents under the age of 18 years bearing a huge variety of psychiatric disorders treated with risperidone or aripiprazole, providing important insights on their safety profile in daily clinical practice.

## Methods

### Data Sources

A descriptive analysis was performed in the EV database on spontaneously reported cases by a healthcare professional (HCP) or non-HCP to a European Union national competent authority or marketing application holder from 2016 through 2018. EV is a centralized European database maintained by the European Medicines Agency of suspected adverse reactions related to medicines authorized in the European Economic Area. Specifically, EV contains all spontaneous individual case safety reports (ICSRs) related to drugs and vaccines. For this analysis, all spontaneous cases associated with risperidone and aripiprazole and referred to the pediatric population aged 3–17 years were selected as aggregate data using the public access online tools in the ADR website, available as line listing and ICSR form. Specifically, the ADR website provides the possibility to retrieve data for the individual cases based on some data elements relative to patient characteristics (age and sex), reactions/events (event, duration, outcome, and seriousness), drugs (role, products or substance, duration of use, dose, units in interval, action taken, indication, pharmaceutical form, and route of administration), and general information such as EV local report number, primary source country, and reporter's qualification (16). All the information as mentioned earlier can be exported in Excel format.

### Data Extraction and Analysis

To perform a descriptive analysis as complete as possible, we obtained the following information reported in both the Excel file and the ICSR form and related to the patient, namely sex (male or female) and age (as this information, as such, is not available in Excel file, it was retrieved from each ICSR), reporter (health professional care or non-health professional care), area of report origin (European Economic Area and Not European Economic Area), reported ADRs, and suspected and concomitant (including interacting) drugs.

Regarding reported ADR, the ICSR reaction section is coded with the Medical Dictionary for Regulatory Activities (MedDRA). MedDRA terms have a hierarchical structure composed of five levels based on the degree of specificity of the term. In ICRSs, all ADRs were coded according to a specific lowest level terms, the first MedDRA hierarchical level. In the present analysis, we categorized all lowest-level terms according to the corresponding system organ class (SOC) level, the highest MedDRA level.

We analyzed the other reported suspected drugs over aripiprazole or risperidone, grouping them according to the first level of the Anatomical Therapeutic Chemical (ATC) classification system. With the same criteria, we analyzed and categorized the concomitant drugs.

Moreover, we analyzed the seriousness of the reported ADR. An adverse reaction is defined as “serious” when it (1) results in death, (2) is life-threatening, (3) requires hospitalization or prolongation of existing hospitalization, (4) results in persistent or significant disability/incapacity (as per reporter's opinion), (5) is a congenital anomaly/congenital disability, or (6) results in some other medically important conditions (http://www.adrreports.eu/it/data_source.html).

To proceed with a clinical evaluation of the reported indications related both to risperidone and aripiprazole, we categorized them based on the Diagnostic and Statistical Manual of Mental Disorders, fifth edition. In addition, we performed a row subanalysis aiming to assess the reported clinical indications in light of what is recommended in the Summary of Product Characteristics (SmPC) of risperidone and aripiprazole. To this end, we have considered the European public assessment reports published by the European Medicines Agency, the SmPC available online on the Italian Medicine Agency website, and also those of the United Kingdom Medicines and Healthcare products Regulatory Agency. More in detail, we analyzed reported clinical indications basing on those recommended in the pediatric population to be able to classify them as “ON Indications” or “OFF Indications” if they were or not included in section 4.1 “Therapeutic indication(s)” within the SmPC.

### Statistical Analysis

A descriptive analysis of all ICSRs related to aripiprazole and risperidone referred to the pediatric population aged 3–17 years during the study period was performed. For continuous variables, descriptive statistics (mean, standard deviation, and frequencies) with percentages were calculated. Comparisons using the Chi-square test and Fisher exact test were performed (significance level was *p* < 0.05) for categorical variables. Data were analyzed using Microsoft Access and Excel programs.

### Compliance With Ethical Standards

Safety data deriving from the EV ADR report online access tool are anonymous and in compliance with the ethical standard. Therefore, no further ethical measures were required (17, 18).

## Results

From 2016 to 2018 in the European Medicine Agency EV database, a total of 3,097 ICSRs were recorded in pediatric patients (3–17 years), of which 855 were for aripiprazole and 2,242 for risperidone, accounting for a total of 11,042 adverse events (AEs) (2,993 for aripiprazole and 8,049 for risperidone), the apparent discrepancy in the number being because single ICSRs can report more than one AE. There was a mean of 3.5 AEs per ICSR for aripiprazole and 3.6 for risperidone. In total, 2,490 (80.4%) ICSRs were related to male patients, specifically 65.0 and 86.3% associated with aripiprazole and risperidone, respectively. Overall, the mean age was of 12 (SD ± 3.5) years, specifically 13 (SD ± 3.6) years for aripiprazole and 11.6 (SD ± 3.4) years for risperidone; the HCP was the main primary source of reports. Moreover, most ICSRs came from non-European economic area for both aripiprazole and risperidone (Table 1).

**Table 1:** Demographic characteristics and distribution for sex, age, seriousness, the type of reporter, country, involving aripiprazole, and risperidone recognized in the EudraVigilance spontaneous reporting system from 2016 to 2018 in pediatric population (3–17 years).

	**Total**	**Aripiprazole**	**Risperidone**
ICSR	3,097	855	2,242
M (%)	2,490 (80.4)	555 (65.0)	1,935 (86.3)
F (%)	578 (18.7)	282 (33.0)	296 (13.2)
Not specified	29 (0.9)	18 (2.1)	11 (0.5)
Mean age, y (±SD)	12 (± 3.5)	13 (±3.6)	11.6 (±3.4)
Overall AEs	11,042	2,993	8,049
AEs serious (%)	10,001 (90.6)	2,425 (81.0)	7,576 (94.1)
N. of AEs per ICSR; Mean (±SD)	3.6 (±2.9)	3.5 (± 3.2)	3.6 (±2.8)
HCP Primary source (%)	2,809 (90.7)	735 (86.0)	2,074 (92.5)
N-HCP Primary source	288 (9.3)	120 (14.0)	168 (7.5)
Country-EEA	830 (26.8)	380 (44.4)	450 (20.1)
Country-NEEA	2,267 (73.2)	475 (55.6)	1,792 (79.9)

*ICSR, individual case safety report; SD, standard deviation; AEs, adverse events; HCP, healthcare professional; N-HCP, non-healthcare professional; EEA, European Economic Area; NEEA, Non-European Economic Area*.

Overall, most of AEs were serious (about 90%); in particular, these were 81.0 and 94.1% for aripiprazole and risperidone, respectively; moreover, in most cases, “other medically important condition” (aripiprazole 48.5%; risperidone 73.8%) and “caused/prolonged hospitalization” (aripiprazole 19.8%; risperidone 12.3%) were the selected seriousness criteria (Table 2).

**Table 2:** Distribution of seriousness criteria selected for the adverse events reported for Risperidone and Aripiprazole.

**Seriousness criteria**	**Aripiprazole**	**Risperidone**	***p***
	***N* (%)**	***N* (%)**	
Other medically important condition	1,175 (48.5)	5,592 (73.8)	*p* < 0.05
Caused/prolonged hospitalization	480 (19.8)	931 (12.3)	*p* < 0.05
Disabling	69 (2.8)	83 (1.1)	*p* < 0.05
Results in death	15 (0.6)	18 (0.2)	*p* < 0.05
Life threatening	13 (0.5)	10 (0.1)	*p* < 0.05
Congenital anomaly	2 (0.1)	0 (0.0)	

Our findings showed that overall “schizophrenia spectrum and other psychotic disorders,” “disruptive, impulse-control, and conduct disorders,” and “autism spectrum disorder” were the top three clinical indications for aripiprazole (19.0, 16.1, and 11.6%, respectively). For risperidone, this analysis showed that “ADHD” (25.4%) followed by “disruptive, impulse-control, and conduct disorders” (17.1%), and “bipolar and related disorders” (14.2%) were the more reported clinical indications (b). Noteworthy, as shown in Table 3, the distribution of the aforementioned clinical indication for aripiprazole and risperidone was significantly changed when we considered the subpopulation aged 3–11 years and those of 12–17 years. In fact, “disruptive, impulse-control, and conduct disorders” (24.3%), “autism spectrum disorder” (17.7%), and “ADHD” (11.9%) were the most reported clinical indication for aripiprazole when used in children aged 3–11 years, whereas “schizophrenia spectrum and other psychotic disorders” (24.3%), followed by “bipolar and related disorders” (12.7%) and “disruptive, impulse-control, and conduct disorders” (12.5%) were the ones observed in children aged 12–17 years. For risperidone, the distribution of clinical indication was almost about similar to that observed for the pediatric population (3–17 years), except for the subpopulation aged 12–17 years, where “schizophrenia spectrum and other psychotic disorders” was the third clinical indication (15.5%) after “ADHD” (25.4%) and “disruptive, impulse-control, and conduct disorders” (17.1%).

**Table 3:** Distribution of reported clinical indication of use for aripiprazole and risperidone.

Reported Clinical Indications^*^	**Aripiprazole** ***N*** **(%)**			**Risperidone** ***N*** **(%)**			***p***
	**3–11 y^#^**	**12–17 y^#^**	**Tot**	**3–11 y^#^**	**12–17 y^#^**	**Tot**	
Schizophrenia spectrum and other psychotic disorders	17 (7.0)	134 (24.3)	151 (19.0)	87 (4.8)	213 (15.5)	300 (9.4)	<0.05
Bipolar and related disorders	15 (6.2)	70 (12.7)	85 (10.7)	249 (13.6)	208 (15.1)	457 (14.2)	<0.05
Disruptive, impulse-control, conduct disorders	59 (24.3)	69 (12.5)	128 (16.1)	334 (18.3)	214 (15.6)	548 (17.1)	<0.05
Depressive disorders	20 (8.2)	62 (11.2)	82 (10.3)	70 (3.8)	96 (7.0)	166 (5.2)	<0.05
Autism spectrum disorder	43 (17.7)	49 (8.9)	92 (11.6)	205 (11.2)	78 (5.7)	283 (8.8)	<0.05
Motor disorders	16 (6.6)	31 (5.6)	47 (5.9)	45 (2.5)	37 (2.7)	82 (2.6)	<0.05
Anxiety disorders	7 (2.9)	24 (4.3)	31 (3.9)	76 (4.2)	63 (4.6)	139 (4.3)	<0.05
ADHD^**^	29 (11.9)	21 (3.8)	50 (6.3)	549 (30.0)	264 (19.2)	813 (25.4)	<0.05
Obsessive-compulsive and related disorders	8 (3.3)	16 (2.9)	24 (3.0)	24 (1.3)	35 (2.5)	59 (1.8)	<0.05
Personality disorders	4 (1.6)	15 (2.7)	19 (2.4)	46 (2.5)	40 (2.9)	86 (2.7)	<0.05
Total Indications	243	552	795	1,828	1,375	3,203	

* Reported clinical indications grouped according to the DSMV;

# y, years;

** *ADHD, attention-deficit and hyperactivity disorders*.

Considering actual recommendations for the pediatric population in terms of clinical indications, results of this analysis suggest that aripiprazole and risperidone were mostly used for clinical indications not reported in the relative SmPC (70.3 and 88.4%, respectively; data not shown). Moreover, it is noteworthy that, irrespective of what is reported in terms of clinical indications specific for pediatric ages, in Europe, aripiprazole is not recommended in children aged <13 years, as reported in the SmPC.

Reported AEs were categorized according to the SOCs; as reported in Table 4, considering the 10 most frequently reported SOC, “psychiatric disorders” (20.2%), “nervous system disorders” (15.9%), and “injury, poisoning, and procedural complications” (14.9%) were the main AEs associated to aripiprazole. “Reproductive system and breast disorders,” followed by “injury, poisoning, and procedural complications” and “psychiatric disorders” were the three top SOCs for risperidone (19.8, 15.5, and 15.0%, respectively). In detail, the most frequently reported AEs related to psychiatric disorders associated with aripiprazole were completed suicide, suicide attempt/ideation, and intentional self-injury, which together accounted for a total of 90 AEs followed by aggression (*N* = 42) and depression, depressed mood, and depressive symptoms (total of 27 AEs) (data not shown). Looking into the reproductive system and breast disorder-related AEs, gynecomastia was the most reported event related to risperidone (*N* = 1,317 out of 1,590 AEs; data not shown).

**Table 4:** Distribution of adverse drug events (AE) categorized according to the system organ classes (SOCs) and involving aripiprazole or risperidone as suspected drugs recognized in EudraVigilance spontaneous reporting system from 2016 to 2018 in pediatric population (3–17 years).

**System organ class**	**Aripiprazole *N* (%)**	**Risperidone *N* (%)**	***p***
Psychiatric disorders	606 (20.2)	1,205 (15.0)	<0.05
Nervous system disorders	476 (15.9)	800 (9.9)	<0.05
Injury, poisoning, and procedural complications	446 (14.9)	1,249 (15.5)	0.4
General disorders and administration site conditions	317 (10.6)	473 (5.9)	<0.05
Investigation	188 (6.3)	549 (6.8)	0.3
Metabolism and nutrition disorders	180 (6.0)	1,042 (12.9)	<0.05
Gastrointestinal disorders	146 (4.9)	142 (1.8)	<0.05
Reproductive system and breast disorders	110 (3.7)	1,590 (19.8)	<0.05
Musculoskeletal and connective tissue disorders	79 (2.6)	117 (1.5)	<0.05
Eye disorders	65 (2.2)	70 (0.9)	<0.05
Total	2,993 (100)	8,047 (100)	

Moreover, as shown in Table 5, SOC distribution for aripiprazole resulted in almost the same even considering the two pediatric subpopulations independently (3–11 years and 12–17 years), although some statistically significant differences were observed for metabolism and nutrition disorders, gastrointestinal disorders, reproductive system and breast disorders, and musculoskeletal and connective tissue disorders. However, SOC distribution for risperidone is varied, considering the two pediatric subpopulations (Table 6). In particular, a part from the reproductive system and breast disorders that were, overall, the most frequently reported SOC, for children aged 3–11 years, metabolism and nutrition disorders followed after that, reaching 18.4% over the total AEs (*N* = 3,901) with a statistically significant difference compared with the 12–17 years subpopulation. Moreover, the most frequently reported AEs related to metabolism and nutrition disorders for risperidone were mainly those related to abnormal weight gain, obesity, and overweight which, accounted for a total of 600 reported AEs (83.6%).

**Table 5:** Distribution of adverse drug events (AEs) categorized according to the system organ classes (SOCs) and involving aripiprazole as a suspected drug, stratified for pediatric patients aged 3–11 and 12–17 years recognized in EudraVigilance spontaneous reporting system from 2016 to 2018.

**System organ class**	**Aripiprazole** ***N*** **(%)**			***p***
	**TOT**	**3–11 years**	**12–17 years**	
Psychiatric disorders	606 (20.2)	191 (18.3)	415 (21.3)	>0.05
Nervous system disorders	476 (15.9)	178 (17.0)	298 (15.3)	>0.05
Injury, poisoning and procedural complications	446 (14.9)	152 (14.6)	294 (15.1)	>0.05
General disorders and administration site conditions	317 (10.6)	106 (10.2)	211 (10.8)	>0.05
Investigation	188 (6.3)	33 (3.2)	155 (8.0)	>0.05
Metabolism and nutrition disorders	180 (6.0)	106 (10.2)	74 (3.8)	<0.05
Gastrointestinal disorders	146 (4.9)	51 (4.9)	95 (4.9)	<0.05
Reproductive system and breast disorders	110 (3.7)	72 (6.9)	38 (1.9)	<0.05
Musculoskeletal and connective tissue disorders	79 (2.6)	27 (2.6)	52 (2.7)	<0.05
Eye disorders	65 (2.2)	12 (1.2)	53 (2.7)	>0.05
Total adverse events	2,993 (100)	1,044 (100)	1,949 (100)	

**Table 6:** Distribution of adverse drug events (AEs) categorized according to the system organ classes (SOCs) and involving risperidone as a suspected drug, stratified for pediatric patients aged 3–11 and 12–17 years recognized in Eudravigilance spontaneous reporting system from 2016 to 2018.

**System organ class**	**Risperidone N (%)**			***p***
	**Tot**	**3–11 years**	**12–17 years**	
Reproductive system and breast disorders	1,590 (19.8)	909 (23.3)	681 (16.4)	<0.05
Injury, poisoning and procedural complications	1,249 (15.5)	635 (16.3)	614 (14.8)	>0.05
Psychiatric disorders	1,205 (15.0)	530 (13.6)	675 (16.3)	<0.05
Metabolism and nutrition disorders	1,042 (12.9)	718 (18.4)	324 (7.8)	<0.05
Nervous system disorders	800 (9.9)	317 (8.1)	483 (11.6)	<0.05
Investigations	549 (6.8)	153 (4.0)	396 (9.6)	<0.05
General disorders and administration site conditions	473 (5.9)	194 (5.0)	279 (6.7)	<0.05
Endocrine disorders	297 (3.7)	150 (3.8)	147(3.5)	>0.05
Gastrointestinal disorders	142 (1.7)	47 (1.2)	95 (2.3)	<0.05
Musculoskeletal and connective tissue disorders	117 (1.5)	30 (0.8)	87 (2.1)	<0.05
Total adverse events	8,047	3,901	4,146	

We also analyzed other suspected drugs reported together with either aripiprazole or risperidone. For aripiprazole, 32% of the ICSRs reported at least another suspected drug, a number reaching 33.5% when risperidone was analyzed. Grouping these other suspected drugs according to the first level ATC classification system, drugs active on the nervous system (ATC N) showed the highest proportion both for aripiprazole (ATC *N* = 83.9%) and risperidone (ATC *N* = 89.0%) (Table 7). Therefore, considering these results, we then analyzed in depth the ATC N group up to the third level, and, as shown in Table 8, antipsychotics (*N* = 250; 44.7% and *N* = 644; 52.5% for aripiprazole and risperidone, respectively) were the most reported other suspected drugs. Moreover, most of the cases reported risperidone, quetiapine, and olanzapine as suspected drugs in addition to aripiprazole and paliperidone, aripiprazole and quetiapine in addition to risperidone. A similar analysis was performed considering also concomitant drugs reported per ICSR (Table 9); once again, as expected, although with a lower proportion than the results described earlier, the ATC-N group was the most frequently reported among the concomitant agents (68.4 and 75% for aripiprazole and risperidone, respectively). Moreover, antidepressants, psychostimulant, and antipsychotic agents were the most frequent ones; however, antidepressants were the most commonly reported concomitant drugs associated to aripiprazole reported as suspected (aripiprazole *N* = 116; 25.6% vs. risperidone *N* = 86; 15.9%); on the other hand, for risperidone, psychostimulants were the most frequently reported agents among concomitant drugs (aripiprazole *N* = 87; 19.2 % vs. risperidone *N* = 108; 19.9%) (Table 10).

**Table 7:** Distribution of other suspected drugs reported with aripiprazole and risperidone grouped according to the Anatomical Therapeutic Chemical (ATC) classification system I level.

ATC^*^	**Aripiprazole *N* (%)**	**Risperidone *N* (%)**	***p***
A	16 (2.4)	22 (1.6)	>0.05
B	2 (0.3)	3 (0.2)	>0.05
C	28 (4.2)	53 (3.8)	>0.05
G	5 (0.8)	3 (0.2)	>0.05
H	8 (1.2)	7 (0.5)	>0.05
J	9 (1.4)	10 (0.7)	>0.05
L	2 (0.3)	7 (0.5)	>0.05
M	6 (0.9)	7 (0.5)	>0.05
N	559 (83.9)	1226 (89.0)	<0.05
P	0 (0.0)	4 (0.3)	–
R	18 (2.7)	27 (2.0)	>0.05
V	6 (0.9)	1 (0.1)	<0.05
UN	7 (1.1)	7 (0.5)	>0.05
TOT	666 (100)	1,377 (100)	

* *ATC, Anatomical Therapeutic Chemical*.

**Table 8:** Distribution of other suspected drugs reported with aripiprazole and risperidone belonging to the Anatomical Therapeutic Chemical (ATC) N group third level: Drugs acting on nervous system.

**ATC N**	**Aripiprazole *N* (%)**	**Risperidone *N* (%)**	***p***
Antipsychotics	250 (44.7)	644 (52.5)	<0.05
Antidepressants	132 (23.6)	182 (14.8)	<0.05
Antiepileptics	62 (11.1)	135 (11.0)	>0.05
Psychostimulants, agents used for Adhd and nootropics	51 (9.1)	142 (11.6)	>0.05
Anxiolytics	25 (4.5)	45 (3.7)	>0.05
Analgesic	16 (2.9)	11 (0.9)	<0.05
Anticholinergic agents	9 (1.6)	21 (1.7)	>0.05
Hypnotics and sedatives	5 (0.9)	13 (1.1)	>0.05
Local anesthetics/anesthetics	3 (0.5)	2 (0.2)	>0.05
Drugs used in addictive disorders	2 (0.4)	4 (0.3)	>0.05
Other nervous system drugs	2 (0.4)	3 (0.2)	>0.05
Anti-dementia drugs	1 (0.2)	5 (0.4)	>0.05
Antiparkinson	1 (0.2)	2 (0.2)	>0.05
Antimigraine	0 (0.0)	17 (1.4)	–
TOT	548 (100)	1,226 (100)	

**Table 9:** Distribution of other concomitant drugs reported with aripiprazole and risperidone grouped according to the Anatomical Therapeutic Chemical (ATC) classification system I level.

ATC^*^	**Aripiprazole *N* (%)**	**Risperidone *N* (%)**	***p***
A	51 (7.7)	34 (4.7)	<0.05
B	7 (1.1)	5 (0.7)	<0.05
C	65 (9.8)	37 (5.1)	<0.05
D	10 (1.5)	1 (0.1)	<0.05
G	4 (0.6)	3 (0.4)	>0.05
H	11 (1.7)	18 (2.5)	>0.05
J	11 (1.7)	25 (3.5)	<0.05
L	3 (0.5)	6 (0,8)	>0.05
M	9 (1.4)	8 (1.1)	>0.05
N	454 (68.4)	542 (75.0)	<0.05
P	0 (0.0)	2 (0.3)	–
R	35 (5,3)	29 (4.0)	>0.05
S	1 (0.2)	1 (0.1)	>0.05
V	3 (0.5)	3 (0.4)	>0.05
UN^**^	0 (0.0)	9 (1.2)	–
TOT	664 (100)	723 (100)	

* ATC, Anatomical Therapeutic Chemical;

** *UN, Unknown*.

*Anatomical Therapeutic Chemical classification system I level Legend*.

*ATC-A, Alimentary Tract and metabolism; ATC-B, Blood and blood-forming organs; ATC-C, cardiovascular system; ATC-D, Dermatologicals; ATC-G, Genito-urinary system and sex hormones; ATC-H, Systemic hormonal preparation, excluding sex hormones, and insulins; ATC-J, antiinfectives for systemic use; ATC-L, Antineoplastic and immunomodulating agents; ATC-M, Muscolo-skeletal system; ATC-N, Nervous system; ATC-P, Antiparasitic products, insecticides, and repellents; ATC-R, Respiratory system; ATC-S, Sensory organs; ATC-V, Various*.

**Table 10:** Distribution of other concomitant drugs reported with aripiprazole and risperidone belonging to the Anatomical Therapeutic Chemical (ATC) N group third level: drugs acting on nervous system.

**Concomitant drug**	**ARI**	**RIS**	***p***
Antidepressants	116 (25.6)	86 (15.9)	<0.05
Psychostimulants, agents used for ADHD And nootropics	87 (19.2)	108 (19.9)	>0.05
Antipsychotics	79 (17.4)	105 (19.4)	>0.05
Antiepileptics	70 (15.4)	96 (17.7)	>0.05
Anxiolytics	46 (7.9)	53 (9.8)	>0.05
Hypnotics and sedatives	30 (6.6)	39 (7.2)	>0.05
Anticholinergic agents	18 (4.0)	20 (3.7)	>0.05
Analgesic	6 (1.3)	12 (2.2)	>0.05
Drugs used in addictive disorders	2 (0.4)	1 (0.2)	<0.05
Anti-parkinson	–	3 (0.6)	–
General anesthetics	–	4 (0.7)	–
Anti-migrane	–	14 (2.6)	–
Local anesthetics	–	1 (0.2)	–
Total	454 (100)	542 (100)	

*ARI, aripiprazole; RIS, risperidone; ADHD, attention-deficit/hyperactivity disorder*.

## Discussion

This study aims to provide a comprehensive overview of all ICSRs spontaneously reported to EV (one of the largest pharmacovigilance databases) in the pediatric population with a huge variety of psychiatric disorders treated with risperidone and aripiprazole, contributing to define their safety profiles in real-world settings.

In line with recent pharmacoepidemiological studies evaluating the safety profile of SGAs in the young population, we found that the largest proportion of ICSRs was reported for boys, and the most frequently reported suspected antipsychotic drug was risperidone (19, 20). These findings are consistent with previous evidence showing that male adolescents are more likely than females to be prescribed atypical antipsychotics and that risperidone is the SGA most commonly prescribed to the young population due to a wider spectrum of indicated uses (21). Despite the higher involvement for risperidone, our results show a similar average of suspected AEs per ICSRs; 3.5 for aripiprazole and 3.6 for risperidone were observed. Also of note, about 90% of overall AEs were serious (81.0 and 94.1% for aripiprazole and risperidone, respectively). Specifically, “other medically important condition” was the most frequently selected seriousness criteria, followed by “caused/prolonged hospitalization” both for aripiprazole and risperidone.

Such a result may have several possible explanations. First, the under-reporting phenomenon that affects especially non-serious AEs is one of the crucial weakness of the spontaneous reporting systems; in fact, it is well-known that clinicians usually focus on serious and medically important events at the expense of the non-serious ones (22, 23). Another possible explanation is that the reporting of non-serious AEs to EV became mandatory only after November 2017. In addition, it could not be ruled out, although it is only speculative in light of the available information, the fact that the frequent use of drugs not in accordance with the SmPC, mostly in terms of clinical indications, is known to increase the severity of AEs; or, that it is suggestive of a more complicated basal clinical condition, considering the number of other suspected and/or concomitant drugs reported, that itself can lead to more serious events (24).

In terms of indication of use, our analysis showed that “schizophrenia spectrum and other psychotic disorders,” “disruptive, impulse-control, and conduct disorders,” and “autism spectrum disorder” were the top three clinical indications for aripiprazole (19.0, 16.1, 11.6%, respectively). For risperidone, this analysis showed that “ADHD” (25.4%) followed by “disruptive, impulse-control, and conduct disorders” (17.1%) and “bipolar and related disorders” (14.2%) were the more reported clinical indications (Table 3).

These findings are consistent with the most recent clinical evidence, indicating that antipsychotics, especially risperidone, are commonly used for the treatment of ADHD, and significantly less for psychoses and disorders of the schizophrenia spectrum, for which, instead, aripiprazole is often the drug of choice (25, 26). ADHD is, in fact, frequently associated with comorbid psychiatric diseases, such as conduct disorders, learning disorders, and tic disorders. In particular, pediatric patients with ADHD and conduct disorders may also suffer from aggressive behavior, leading to antipsychotic drug use. Indeed, clinical evidence suggests that risperidone, combined with methylphenidate, represents an appropriate pharmacological approach effective for controlling both the aggression and the core symptoms of the ADHD (25, 26). Another finding emerging from our results is the use of aripiprazole and risperidone for bipolar disorders. This finding may be interpreted in the light of a significant increase of clinical trial in the pediatric setting, the results of which are intended as clinical evidence able to guide in choosing the more appropriate drug in psychiatric children, a therapeutic area with high unmet therapeutical need due to the scarce number of drugs approved (27–29). In line with that, as for risperidone, also aripiprazole is now included in the list of drugs of the Italian law 648/1996 for manic episodes associated with bipolar I disorder in pediatric patients (≥10 years) (30). As shown in Table 3, significant differences were observed between pediatric subpopulations aged 3–11 and 12–17 years, in terms of clinical indications, especially for aripiprazole. Our results, in fact, suggest that aripiprazole is mainly used for disruptive, impulse-control, and conduct disorders in children and for schizophrenia spectrum and other psychotic disorders in adolescents; this finding could correlate with the different time of onset of the two conditions, being schizophrenia diagnosed in the developmental age of emerging adulthood (31) and disruptive, impulse-control, and conduct disorders, most often in late childhood or the early teen years (32, 33). Using the classification of SOCs, we found that psychiatric reactions and reproductive system and breast disorders were the most frequently reported AEs for aripiprazole and risperidone, respectively. These results are similar to those of Jakobsen KD and collaborators; they analyzed ADRs concerning aripiprazole-associated neurological and psychiatric events in children and adolescents through the database of the Danish Medicines Agency and observed that, in patients with psychotic disorders, aripiprazole could lead to aggressive behavior, anxiety, hallucinations, mental tics, neuroleptic malignant syndrome, overeating, and suicidal behavior (34, 35). Our findings also reveal that aggression was one of the most frequently reported psychiatric aripiprazole-induced event; moreover, we observed several ICSRs of suicidal behavior, such as completed suicide, suicide attempt/ideation, and intentional self-injury; these accounted for a total of 90 AEs. There is little information on suicidal behavior due to antipsychotics, especially in a pediatric setting. This is probably due to the difficulty of considering this AE separately from the underlying pathology that made treatment with antipsychotics necessary. On the one hand, suicidal attempt, suicidal ideation, or suicide completed are all reported in the European SmPC relative to aripiprazole, albeit with unknown incidence. These results highlight the need to closely monitor these serious AEs to better define this potential risk, especially in pediatric patients.

On the other hand, the scientific literature has been enriched over time of cases of worsened psychotic symptoms either in subjects in which aripiprazole was an add-on antipsychotic drug or in patients switched to aripiprazole. In this regard, it has been hypothesized, especially in schizophrenic patients, the occurrence of hypersensitivity to the agonistic effects of aripiprazole, which exert its effects through partial dopamine-2 (D2) receptor agonism, after treatment with traditional antipsychotics acting as dopamine antagonist (36, 37). Considering the nature and quality of our dataset, it was not possible to investigate each ICSR from a clinical and therapeutic point of view; however, the high number of cases of suicidal behavior cannot be ignored, and it certainly suggests careful monitoring of clinicians in children and adolescents receiving aripiprazole.

Regarding the incidence of breast abnormalities, as expected, we found a very common incidence with risperidone. Dopamine acts by inhibiting prolactin; therefore, antipsychotic medications, blocking the D2 receptor via the tuberoinfundibular pathway, lead to hyperprolactinemia. Consequences of having high prolactin levels include galactorrhea and gynecomastia in female and male patients, respectively (38).

Our analysis suggests that AEs related to metabolism and nutrition disorders associated with risperidone were more frequently reported in children than adolescents. Actually, we found that weight gain was the most frequently reported AE related to risperidone. This finding is also in line with scientific evidence that, among SGAs, risperidone could cause significant weight gain, especially in children (39, 40). Although younger age, together with male sex and low basal body mass index, as predictor factor antipsychotic-related weight gain, is still debated among the scientific community, several studies are suggesting that children are at higher risk for this AE and in general for risperidone-related AEs (41, 42). The risperidone-related weight gain that we have observed especially in children rather than in adolescents, although it could not be better clinically assessed, highlights the need of monitoring not only efficacy, but especially adverse effects, focusing on those related to metabolism and nutrition disorders, to periodically evaluate the benefit–risk ratio.

Finally, our analysis highlights that both among other suspected drugs and among concomitant ones, antipsychotics were the most frequently reported. More in depth, risperidone, quetiapine, and olanzapine were the active drugs reported as suspected in addition to aripiprazole and paliperidone, aripiprazole and quetiapine in combination with risperidone. These findings are in line with recent data showing that also in children and adolescents, antipsychotic polypharmacy is increasing (42, 43). The concern here is that in this population, there is still little evidence on efficacy and, especially, tolerability of such clinical practice (43); indeed, contrary to what has been demonstrated in the adult population, the use of more than one antipsychotic concomitantly could only increase the risk of ADRs occurrence, negatively impacting on adherence and quality of life and increasing healthcare costs. A recent cohort study with up to 20-year follow-up carried out in the adult population on 62,250 patients with schizophrenia showed that patients had the lowest risk of psychiatric or all-cause hospitalization when they administered antipsychotic polypharmacy, especially the combination therapy with clozapine plus aripiprazole. This study also suggested that antipsychotic polypharmacy may be more effective for maintenance treatment compared with a monotherapy regimen (44). Saldaña et al. recently published a retrospective study carried out on 1,427 children and adolescents who were consecutively admitted and discharged from the psychiatry service of an urban hospital. Of 1,427 enrolled patients, 58.9% were prescribed one or more antipsychotics at discharge, and, of these, 13.8% were discharged with antipsychotic polypharmacy. Similar to what we have observed, the most frequent combinations were aripiprazole/quetiapine, risperidone/quetiapine, aripiprazole/risperidone, and olanzapine/quetiapine. Through multivariate logistic regression, the authors have identified potential predictor factors associated with antipsychotic polypharmacy, suggesting that, overall, more severe illness and treatment resistance played an important role in this therapeutic strategy (45). In a line similar to that of Saldaña et al. our analysis shows the common use of antipsychotics polypharmacy often associated with serious suspected ADRs leading to hospitalization.

## Strengths and Limitations

The present study, based on data from a spontaneous reporting system, has several strengths. First and foremost, it is one of the largest databases of a spontaneous report of ADR; secondly, it allows us to take a picture of the actual tolerability profile of widely used drugs, such as risperidone and aripiprazole in children and adolescent. Moreover, data based on spontaneous reporting ADRs unmasked previously unidentified safety signals that deserve further and specific studies and draw the attention of clinicians and regulatory authorities to the need to continue monitoring the incidence of serious AEs, such as, for example, the suicidal behavior that we have observed associated with aripiprazole. Moreover, studies based on spontaneous reporting systems can also confirm statistically significant safety issues already known, such as antipsychotic-related weight gain, stressing, once again, the need for much closer patients monitoring. Finally, data from the spontaneous reporting systems can also offer a much closer approach to the daily clinical practice of using drugs and therefore suggest potential inappropriate uses, which may be of concern, especially for the pediatric population. The present study also has several limitations. Firstly, analyzing a large database of spontaneous reporting systems is not always the proper method to assess tolerability issues more complex than a specific AE. In fact, aggregate data available through the public access online tools ADR website are mainly useful for descriptive analysis, the interpretation of which is difficult from a clinical point of view. Secondly, with regard to the indications of use, our data seem to suggest a greater proportion of uses not reported in the SmPC of risperidone and aripiprazole specifically for the pediatric population; although these results are in line with those of several naturalistic studies, we are, on the other hand, unable to assert with certainty that all of these cases were of off-label uses.

Finally, this study also shares the limitations that usually characterize most of the studies based on data of spontaneous reporting ADRs, namely underreporting, information quality, often scant, and lack of denominator data, that is, the actual drug exposed (46).

## Conclusion

The present analysis provides an overview of all spontaneously reported ICSRs in the EV database, from 2016 to 2018, in children and adolescents treated with risperidone and aripiprazole. Overall results demonstrate that spontaneously reported AEs associated with aripiprazole and risperidone reflect what is already known in terms of safety profile, although ~90% of them being serious and in 19.8 and 12.3% of these related to aripiprazole and risperidone, respectively, hospitalization was required. Our results demonstrate the widespread use of aripiprazole and risperidone with clinical indications not approved for the pediatric population. Based on these findings, we conclude that it is very important for competent authorities together with international scientific societies to promote pediatric investigation plans, effective training, and information initiatives to confirm the actual efficacy of these drugs in pediatric populations, thus reducing harm to these patients, improving public health and reduce healthcare costs.

## Data Availability Statement

The datasets generated for this study will not be made publicly available. The distribution of european pharmacovigilance data needs the authorization from the European Medicine Agency except for aggregated data available at Adrreports.eu portal (http://www.adrreports.eu/it/search.html).

## Ethics Statement

Ethical review and approval was not required for the study on human participants in accordance with the local legislation and institutional requirements. Written informed consent from the participants' legal guardian/next of kin was not required to participate in this study in accordance with the national legislation and the institutional requirements.

## Author Contributions

CR, MGS, CC, MP, BS, SR, RB, FR, EC, and AC: drafting the work and revising it for important intellectual content, final approval of the version to be published, and agreement to be accountable for all aspects of the work in ensuring that questions related to the accuracy or integrity of any part of the work are appropriately investigated and resolved. CR, MGS, and CC: substantial contributions to the acquisition, analysis, or interpretation of data for the work, and wrote the paper. EC and AC: developed the concept and designed the study. All authors contributed to the article and approved the submitted version.

## Conflict of Interest

The authors declare that the research was conducted in the absence of any commercial or financial relationships that could be construed as a potential conflict of interest.
